# Bergenin ameliorates cognitive deficits and neuropathological alterations in sodium azide-induced experimental dementia

**DOI:** 10.3389/fphar.2022.994018

**Published:** 2022-09-29

**Authors:** Rajeev K. Singla, Konika Dhonchak, Rupinder K. Sodhi, M. Arockia Babu, Jitender Madan, Reecha Madaan, Suresh Kumar, Rohit Sharma, Bairong Shen

**Affiliations:** ^1^ Institutes for Systems Genetics, Frontiers Science Center for Disease-Related Molecular Network, West China Hospital, Sichuan University, Chengdu, Sichuan, China; ^2^ School of Pharmaceutical Sciences, Lovely Professional University, Phagwara, Punjab, India; ^3^ Department of Pharmacology, Chandigarh College of Pharmacy, Mohali, India; ^4^ National Institute of Pharmaceutical Education and Research, Hyderabad, India; ^5^ Chitkara College of Pharmacy, Chitkara University, Rajpura, Punjab, India; ^6^ Department of Pharmaceutical Sciences and Drug Research, Punjabi University, Patiala, Punjab, India; ^7^ Department of Rasa Shastra and Bhaishajya Kalpana, Faculty of Ayurveda, Institute of Medical Sciences, Banaras Hindu University, Varanasi, Uttar Pradesh, India

**Keywords:** dementia, Alzheimer’s disease, bergenin, PPAR-γ, BADGE, memory, oxidative stress, neuroinflammation

## Abstract

**Background:** Bergenin, 4-O-methyl gallic acid glucoside, is a bioactive compound found in the cortex of Mallotus japonicus (L.f.) Müll.Arg. along with many other natural resources including that from Bergenia species. The present study delineates the neuroprotective potential of bergenin through the modulation of PPAR-γ receptors.

**Method:** Dementia was induced in the Wistar rats by intraperitoneal (i.p.) administration of sodium azide (12.5 mg/kg for the first 5 days followed by 10 mg/kg for the next 9 days). The rats were then exposed to the Morris water maze test to assess the effect on cognitive abilities followed by a series of biochemical and histopathological evaluations.

**Results:** Sodium azide-treated rats exhibited a severe deterioration of memory as suggested by poor performance in the spatial learning task in addition to the enhancement of brain acetylcholinesterase potential, oxidative stress, inflammation, and amyloid-β (Aβ) accumulation. Administration of bergenin to sodium azide-treated rats significantly recovered cognition and related biochemical variations. Further, co-administration of Bisphenol A diglycidyl ether (BADGE), a PPAR-γ antagonist with bergenin challenged its neuroprotective effects.

**Conclusions:** The findings of our study exhibit that the cognitive restoration potential of bergenin may be attributed to its modulatory effects against cholinesterase, oxidative stress, and inflammatory markers, as well as its neuroprotective actions, thus aligning it as a possible therapy for Alzheimer’s disease-related dementia. The study also fortifies the significance of PPAR-γ receptors in dementia.

## Graphical Abstract

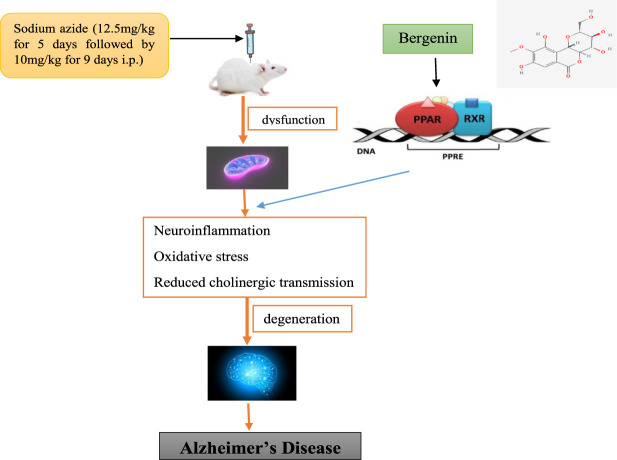

## 1 Introduction

Dementia is a set of symptoms caused by several neurological disorders and among which Alzheimer’s disease (AD) is the most frequent cause, accounting for 50%–70% of cases (Ping, 2015; Hampel et al., 2018). AD is an advanced neurodegenerative disorder that interferes with the person’s daily performance like difficulties in speaking, problem-solving, and other cognitive skills, along with changes in mood and behavior due to memory and neuronal loss (Moussa-Pacha et al., 2019). The pathogenesis of AD includes the accumulation of amyloid- β (Aβ) protein in the form of extracellular senile plaques and intracellular neurofibrillary tangles formed by tau protein fibrils causing synaptic loss and neurodegeneration which leads to memory impairment and other cognitive problems (Weiner et al., 2013). Aβ causes induction of calcium-dependent excitotoxicity as it is correlated with the development of reactive oxygen species (ROS) as well as reactive nitrogen species (RNS). It also impairs cellular respiration and alters the synaptic functions which are associated with cognitive deficiencies (Butterfield et al., 2014). Oxidative stress begins early during AD and is among the major molecular change in the pathogenesis of AD as demonstrated by numerous studies (Gao et al., 2017; Beg et al., 2018). The current treatments which are available for AD include acetylcholinesterase inhibitors viz. memantine clinically used for the treatment and management of patients having moderate-to-severe AD (Weller and Budson, 2018). Diagnosis of AD is a difficult process and requires the patient to go through many blood tests, physical examinations, psychiatric evaluations, and brain scans like CT, MRI, and PET. Before the early 2000s, an autopsy was done after the death of the individual to know whether a person had Alzheimer’s disease. Now with the progressive research and advances in technology, the preclinical diagnosis of AD can be done by recent non-invasive imaging techniques using Aβ- and tau-positron emission tomography (PET) tracers which allow the tracking of the evolution of AD during the patient’s lifetime (Sáez-Orellana et al., 2020).

Sodium azide is a colorless crystalline solid and is a highly toxic substance appropriate for fabricating AD-like indications in rodents and for evaluating neuroprotective agents. Sodium azide is a selective inhibitor of the cyclooxygenase (COX) enzyme that induces a reduction in the activity of complex IV of the electron transport chain (ETC) and thus causes mitochondrial dysfunction which further leads to memory deficits (Weinstock and Shoham, 2004). Peroxisome proliferator-activated receptors (PPARs) are ligand-activated transcription factors that contribute a major function in the regulation of glucose assimilation and the homeostasis of lipid metabolism. Further, they are also known to be related to repressing the expression of genes related to inflammation (Lecarpentier et al., 2017). The results of clinical and animal studies exhibit that the utilization of Peroxisome proliferator-activated receptor gamma (PPAR-γ) agonist improves both learning and memory with other AD-related pathology. Therefore, these receptor agonists represent a beneficial therapeutic target for AD (Khan et al., 2019). Bergenin act as a PPAR-γ agonist (Xiang et al., 2020) and has antioxidative (Qiao et al., 2019), anti-inflammatory activity (Lim et al., 2000), BACE1 inhibitory activity (Madaan et al., 2022), and modulation of Nrf-2/NF-κB Pathway (Shal et al., 2021). Bergenin treatment restored the actions of ETC, particularly complex I, complex II, and complex IV; curtailed the lipid peroxidation; scavenged enhanced reactive oxygen species, and up-regulated antioxidant levels leading to the amelioration of mitochondrial dysfunction (Aggarwal et al., 2016). Bisphenol A diglycidyl ether (BADGE) is a synthetic substance and has PPAR-γ antagonistic activity (Dworzanski et al., 2010).

## 2 Materials and methods

### 2.1 Laboratory animals

Wistar rats of either sex (180–220 g) 8–10 weeks of age (NIPER, Punjab, India) were employed in the present study. Standard laboratory feed, procured from Ashirwad Industries, Punjab, India, and water ad libitum were provided to the animals exposed to 12 h light and dark cycle. The experimental protocol (CCP/IAEC/Feb 2021/9) was approved by the Institutional Animal Ethics Committee (IAEC) (Reg no. 1201/PO/Re/S/CPCSEA) and as per the standard guidelines given by CPCSEA, India.

### 2.2 Drugs and reagents

The materials used in the experiments were of analytical quality grade and were freshly prepared before use. Sodium azide (SA) was purchased from Nice Chemicals (P) Ltd., India. Bergenin (≥98.0%, HPLC, analytical standard) and Bisphenol A diglycidyl ether (BADGE, ≥95.0%, HPLC, analytical standard) were procured from Sigma Aldrich, India.

### 2.3 Laboratory models

#### 2.3.1 Sodium azide-induced dementia

Dementia was produced in Wistar rats by intraperitoneal administration of sodium azide 12.5 mg/kg for the initial 5 days followed by 10 mg/kg for the next 9 days (Virdi et al., 2020).

#### 2.3.2 Morris water maze test

In the present study, the learning and memory of the animals were assessed using the Morris water maze test (Morris, 1984; Bhatia et al., 2021). Morris water maze (MWM) is a swimming-based exteroceptive model in which a hidden platform is positioned and the animal learns to escape onto it (Sodhi and Singh, 2014). Animals were subjected to training for four successive days and the starting position was altered with each exposure (as shown in Table 1) (Kaur and Sodhi, 2015). On day 4 of escape latency time (ELT), the time spent exploring the concealed platform in the water maze, was recorded as an index of acquisition/learning. The platform was then detached on the fifth consecutive day and the mean time disbursed in altogether four quadrants was noted as an index of the acquisition of memory (Jain and Sharma, 2016). In the search for the concealed platform, the total time spent in the target quadrant (TSTQ) by the animal on the fifth day was noted as an indicator of recovery of memory.

**TABLE 1:** Pattern of training trails on MWM test. (Q refers to quadrant). This table was adopted from our previous work (Kaur and Sodhi, 2015).

Day 1	Q1	Q2	Q3	Q4
Day 2	Q2	Q3	Q4	Q1
Day 3	Q3	Q4	Q1	Q2
Day 4	Q4	Q1	Q2	Q3

### 2.4 Biochemical parameters estimations

#### 2.4.1 Collection of samples

The laboratory animals were sacrificed by cervical displacement after the completion of the planned experiment (Sodhi and Singh, 2013a). Brains were then removed, followed by homogenization with a phosphate buffer of pH 7.4. Centrifugation of the samples was then done at 3,000 rpm for 15 min (Bansal and Parle, 2010). The supernatant was then used for further experiments, related to acetylcholinesterase, total brain protein, reduced glutathione levels, nitrate/nitrite levels, thiobarbituric acid, myeloperoxidase activity, and pro-inflammatory cytokines.

#### 2.4.2 Assessment of brain acetylcholinesterase activity

The total-brain acetylcholinesterase activity was evaluated as per the protocol given by (Ellman et al., 1961) with slight modifications as done by (Koladiya et al., 2009; Sain et al., 2011; Bhatia and Singh, 2020).

#### 2.4.3 Assessment of brain thiobarbituric acid reactive species level

The quantitative measurement of thiobarbituric acid reactive species (TBARS) was carried out according to the method of Niehaus and Samuelsson (Niehaus and Samuelsson, 1968; Gulati and Singh, 2014). The absorbance was measured at 535 nm against a blank reagent. The values were expressed as nanomoles per mg of protein (Sharma and Singh, 2012).

#### 2.4.4 Assessment of reduced glutathione level

Reduced glutathione (GSH) content of brain tissue was estimated using a method given by Beutler (Beutler et al., 1963; Sharma and Singh, 2012). The absorbance was measured spectrophotometrically at 412 nm. The results were expressed as micromoles of reduced glutathione per mg of protein (Sodhi and Singh, 2013a).

#### 2.4.5 Assessment of brain myeloperoxidase level

The myeloperoxidase (MPO) activity was estimated as per the protocol given by Krawisz (Krawisz et al., 1984; Sodhi and Singh, 2013a).

#### 2.4.6 Estimation of pro-inflammatory cytokines

The pro-inflammatory cytokines viz. TNF-α and IL-1β were measured with standard ELISA kits and the procedure adopted was that provided by the company (R & D Systems, Minneapolis, United States).

#### 2.4.7 Assessment of brain nitrite/nitrate level

The brain nitrite/nitrate level was estimated as per the procedure given by Sastry and the team (Sastry et al., 2002) using a colorimetric assay. The results were articulated as micrograms per mg of protein.

#### 2.4.8 Histopathological assessment

##### 2.4.8.1 Hematoxylin and Eosin staining

Finally, brain tissue was fixed in 4% formalin to avert autolysis and putrefaction. Tissue processing was made following the standard procedures of fixation, dehydration, impregnation, embedding, sectioning, and staining with hematoxylin and eosin by the method of Bancroft (Bancroft, 2019). The micrographs of stained sections (5-μm thickness) were consequently visualized using a light microscope (×40) (Sodhi and Singh, 2013b).

##### 2.4.8.2 Congo red staining

The brain sections (5-μm thickness) were kept in Congo red solution for 18 min (Sodhi and Singh, 2013a). Subsequently, the sections were washed with water for 20 min and then immersed in a weak base for 10 s following which the brain sections were dipped in hematoxylin for 5 min, and again cleaned with flowing water (Sodhi and Singh, 2013a; Sodhi and Singh, 2013b; Puchtler et al., 2017). The Aβ′s accumulation was studied through a light microscope (×100) (Sodhi and Singh, 2013a).

### 2.5 Experimental protocol

**FIGURE 1:**
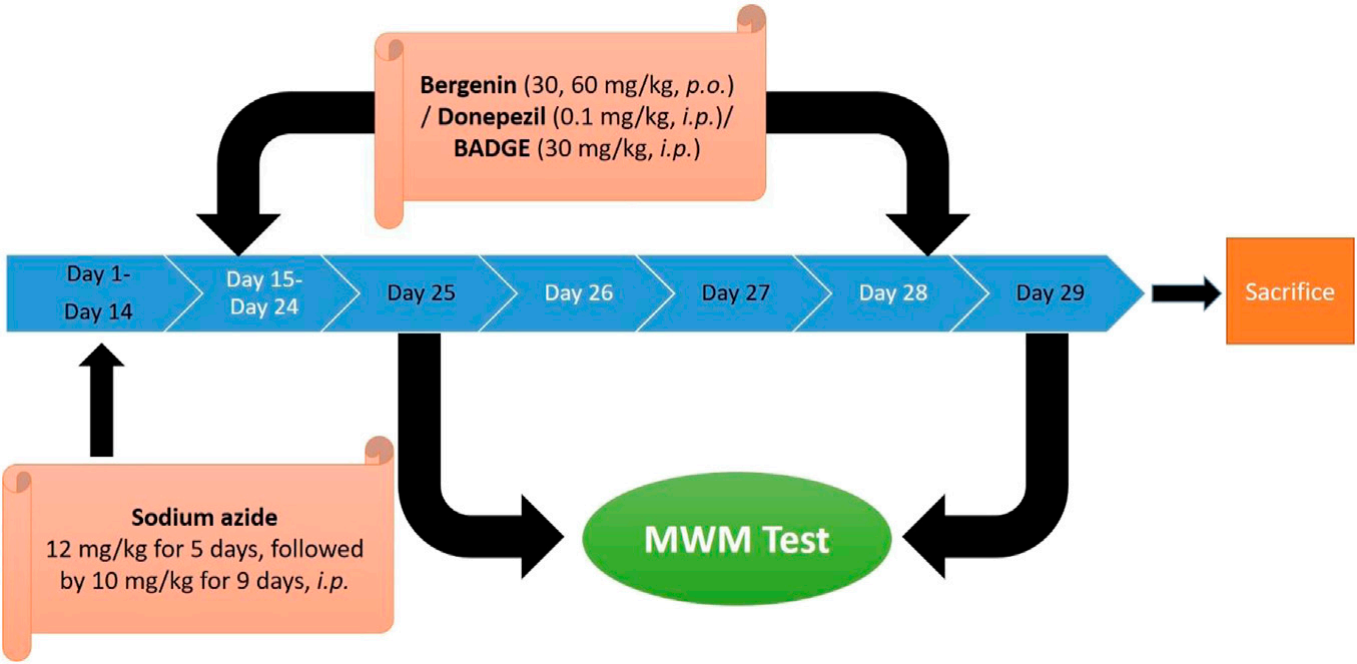
Experimental protocol.

The study comprised nine separate investigational groups each consisting of 6 Wistar rats. The animals were administered sodium azide (12.5 mg/kg for the first 5 days followed by 10 mg/kg. for 9 days i.p, for 2 weeks). The drug treatment (donepezil, 0.1 mg/kg/day, i.p /bergenin, 30 mg/kg and 60 mg/kg p.o. /BADGE 30 mg/kg i.p.) was administered for 14 days starting from day 15 up to day 28. The procedure adopted was the same as performed in our previous study (Sodhi and Singh, 2013a) (Figure 1; Table 2).

**TABLE 2:** Details of different treatment groups.

S.No	Group	Treatment
1	Normal control	Untreated
2	DMSO	10 ml/kg, p.o.
3	SA control	Sodium azide 12.5 mg/kg for first 5 days followed by 10 mg/kg for 9 days i.p
4	BER per se	Bergenin 30 mg/kg p.o. for 14 days
5	SA + Donepezil	(Sodium azide 12.5 mg/kg for first 5 days followed by 10 mg/kg. for 9 days i.p) + (Donepezil 0.1 mg/kg i.p. for 14 days)
6	SA + BER (LD)	(Sodium azide 12.5 mg/kg for first 5 days followed by 10 mg/kg. for 9 days i.p)+ (Bergenin 30 mg/kg p.o. for 14 days)
7	SA + BER (HD)	(Sodium azide 12.5 mg/kg for first 5 days followed by 10 mg/kg. for 9 days i.p) + (Bergenin 60 mg/kg p.o. for 14 days)
8	SA + BADGE	(Sodium azide 12.5 mg/kg for first 5 days followed by 10 mg/kg. for 9 days i.p) + (BADGE 30 mg/kg i.p. for 14 days)
9	SA + BER (LD) + BADGE	(Sodium azide 12.5 mg/kg for first 5 days followed by 10 mg/kg. for 9 days i.p) + (Bergenin 30 mg/kg p.o. for 14 days) + (BADGE 30 mg/kg i.p. for 14 days)

“DMSO, dimethyl sulfoxide; SA, sodium azide; BER, bergenin; BER (LD), bergenin low dose; BER (HD), bergenin high dose; BADGE, Bisphenol A diglycidyl ether” (Mehra et al., 2015).

### 2.6 Statistical evaluation

The results obtained were expressed as mean ± Standard error of the mean (S.E.M). The data from various groups were statistically examined using a one-way analysis of variance (ANOVA) followed by Bonferroni’s multiple range test. The *p* <0.05 was considered to be statistically significant (Kaur and Sodhi, 2015).

## 3 Results

### 3.1 Effect on cognitive parameters using Morris water maze

**FIGURE 2:**
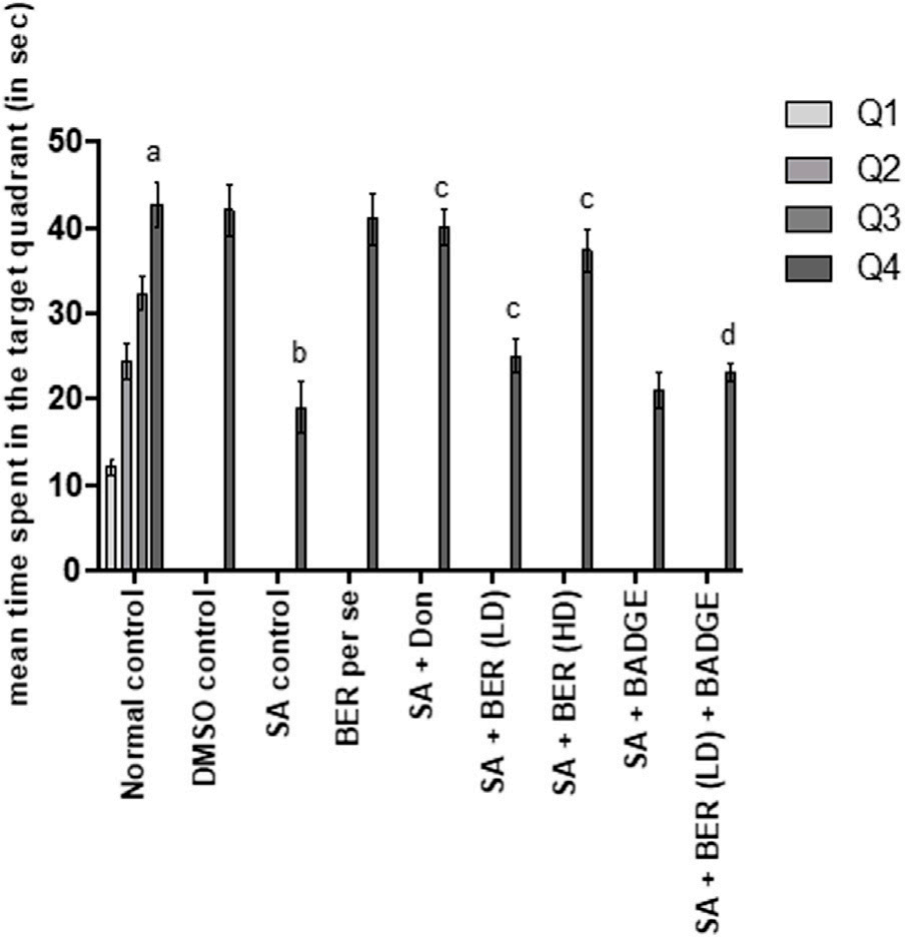
Effect of pharmacological interventions on the total time spent in the target quadrant (TSTQ) in seconds using the Morris water maze test. “DMSO, dimethyl sulfoxide; SA, sodium azide; Don, donepezil; BER, bergenin; BER (LD), bergenin low dose; BER (HD), bergenin high dose; BADGE, Bisphenol A diglycidyl ether” (Mehra et al., 2015). “Each group (*n* = 6) represents mean ± S.E.M, a = *p* ˂ 0.05 vs. time spent in quadrant one in normal control, b = *p* ˂ 0.05 vs. time spent in the target quadrant in normal control, c = *p* ˂ 0.05 vs. time spent in the target quadrant in SA control, d = *p* ˂ 0.05 vs. time spent in target quadrant in SA + BER (LD) treated group” (Sodhi and Singh, 2013b). One-way ANOVA followed by Bonferroni’s multiple range test.

The results indicated that control animals exhibited a significant reduction in day 4 ELT in comparison to day 1 ELT (Kaur and Sodhi, 2015). Furthermore, during the retrieval trial on day 5 of the MWM test, it was observed that the animals spent more time in the target quadrant (Q4) in the search for the missing platform versus the total time spent in other quadrants (Q1, Q2, and Q3) suggestive of learning and memory (Kaur and Sodhi, 2015). The treatment with sodium azide significantly prevented the decline in day 4 ELT in comparison to the control group (Table 3) (Sodhi and Singh, 2013b). A marked diminution was also indicated in TSTQ (Q4) in the recovery trial on the fifth day (Figure 2). Further, change on the fourth day of ELT was not statistically significant (Table 3) and on day 5 TSTQ (Figure 2) was indicated in bergenin (30 mg/kg/day, p.o.) [BER Low-Dose (LD)] per se group in comparison to the control group (Sodhi and Singh, 2013a). In a dose-dependent way, co-administration of donepezil (0.1 mg/kg/day, i.p.) along with bergenin (30 mg/kg and 60 mg/kg p.o. [BER High-Dose (HD)] potentially decreases the fourth-day rise in ELT (Table 3) and a decline in fifth-day TSTQ in sodium azide-treated rats (Figure 2) (Sodhi and Singh, 2013a). Furthermore, co-administration of BADGE (30 mg/kg i.p.) with bergenin (30 mg/kg p.o.) antagonized the effect produced by the bergenin dose (Table 3; Figure 2) (Sodhi and Singh, 2013a). These results are aligning with our previous studies related to rifampicin and all-trans retinoic acid (Sodhi and Singh, 2013a; Kaur and Sodhi, 2015).

**TABLE 3:** “Effect of pharmacological interventions on escape latency time (ELT) on day 1 and day 4 (time in seconds) using Morris water maze (MWM) test in sodium azide-treated rats.” Performed in a way similar to our previously published work (Kaur and Sodhi, 2015).

Group	Treatment	Dose	ELT-Day 1 (sec)	ELT-Day 4 (sec)
I	Normal control	Untreated	114 ± 1.15	62.5 ± 2.65^a^
II	DMSO	10 ml/kg, p.o.	112 ± 1.30	62.8 ± 1.42
III	SA control	12.5 mg/kg i.p. 5 days and 10 mg/kg i.p. for 9 days	113.3 ± 1.70	94.5 ± 1.23^b^
IV	BER per se	30 mg/kg p.o.	106 ± 1.68	60 ± 1.99
V	SA + Donepezil	(12.5 mg/kg i.p. 5 days and 10 mg/kg i.p. for 9 days) + (0.1 mg/kg i.p.)	105 ± 1.98	65 ± 2.01^c^
VI	SA+ BER (LD)	(12.5 mg/kg i.p. 5 days and 10 mg/kg i.p. for 9 days) + (30 mg/kg p.o.)	110 ± 1.31	77.1 ± 0.79^c^
VII	SA + BER (HD)	(12.5 mg/kg i.p. 5 days and 10 mg/kg i.p. for 9 days) + (60 mg/kg p.o.)	102.5 ± 1.23	69.1 ± 2.91^c^
VIII	SA + BADGE	(12.5 mg/kg i.p. 5 days and 10 mg/kg i.p. for 9 days) +30 mg/kg i.p.	109.4 ± 1.05	92 ± 1.78
IX	SA + BER (LD) + BADGE	(12.5 mg/kg i.p. 5 days and 10 mg/kg i.p. for 9 days) + (30 mg/kg p.o.) + (30 mg/kg i.p.)	108.1 ± 1.57	90 ± 2.38^d^

“DMSO, dimethyl sulfoxide; SA, sodium azide; BER, bergenin; BER (LD), bergenin low dose; BER (HD), bergenin high dose; BADGE, Bisphenol A diglycidyl ether” (Mehra et al., 2015). “Each group (*n* = 6) represents mean ± S.E.M, a = *p* < 0.05 as compared to the day 1 ELT in control, b = *p* < 0.05 as compared to the day 4 ELT in control, c = *p* < 0.05 as compared to the day 4 ELT in SA control, d = *p* < 0.05 as compared to the day 4 ELT in SA + BER (LD) treated group.”

### 3.2 Impact on brain acetylcholinesterase activity

**FIGURE 3:**
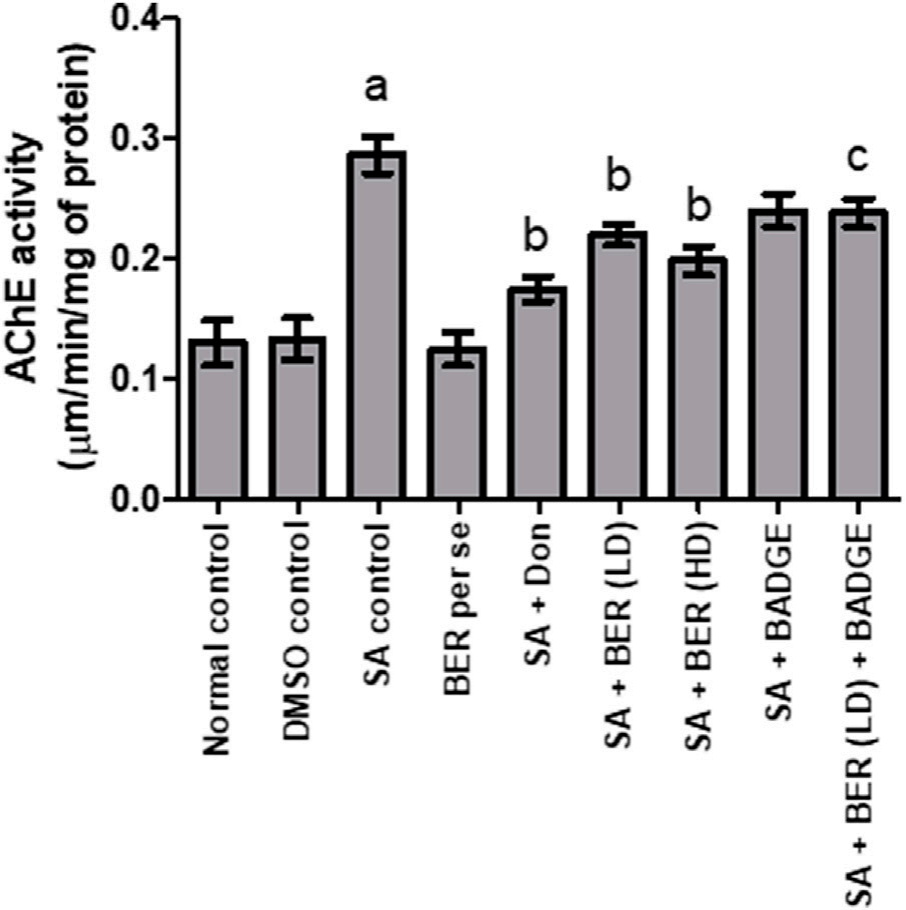
Effect of pharmacological interventions on brain AChE activity (μM of ACh hydrolyzed/min/mg protein). “DMSO, dimethyl sulfoxide; SA, sodium azide; Don, donepezil; BER, bergenin; BER (LD), bergenin low dose; BER (HD), bergenin high dose; BADGE, Bisphenol A diglycidyl ether” (Mehra et al., 2015). “Each group (*n* = 6) represents mean ± S.E.M, a = *p* ˂ 0.05 vs. normal control, b = *p* ˂ 0.05 vs. normal control, c = *p* ˂ 0.05 vs. SA control. One way ANOVA followed by Bonferroni’s multiple range test” (Gulati and Singh, 2014).

Sodium azide-treated rats have suggestively boosted the brain acetylcholinesterase (AChE) activity in contrast to the rats from the control set (Figure 3) (Sodhi and Singh, 2013a). Bergenin per se (30 mg/kg p.o.) had no significant impact on brain AChE activity when compared with the control (Figure 3). Administration of donepezil and bergenin to sodium azide treated rats potentially decrease brain AChE activity while comparing with the control group (Figure 3). Co-administration of bergenin (30 mg/kg p.o.) along with BADGE (30 mg/kg i.p.) to sodium azide treated rats markedly enhanced brain AChE activity (Figure 3).

### 3.3 Impact on brain’s thiobarbituric acid reactive species and brain’s glutathione levels

**FIGURE 4:**
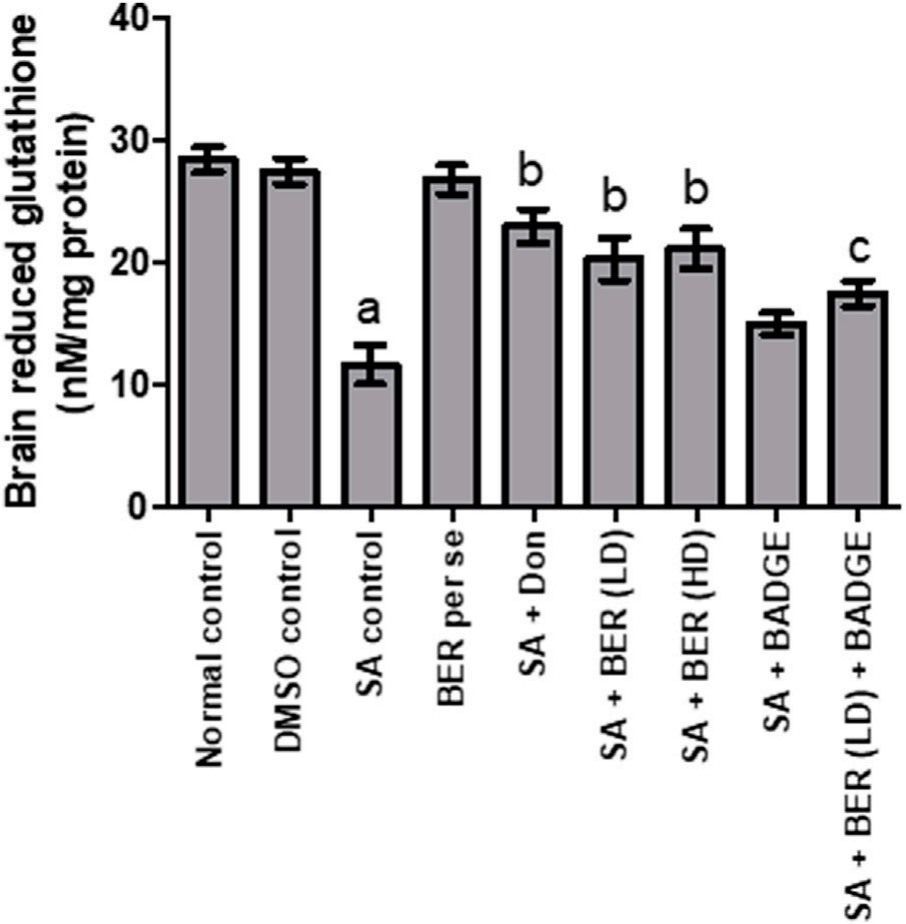
Effect of pharmacological interventions on brain GSH levels (μM/mg of protein). “DMSO, dimethyl sulfoxide; SA, sodium azide; Don, donepezil; BER, bergenin; BER (LD), bergenin low dose; BER (HD), bergenin high dose; BADGE, Bisphenol A diglycidyl ether” (Mehra et al., 2015). “Each group (*n* = 6) represents mean ± S.E.M, a = *p* ˂ 0.05 vs. normal control, b = *p* ˂ 0.05 vs. normal control, c = *p* ˂ 0.05 vs. SA control. One way ANOVA followed by Bonferroni’s multiple range test” (Gulati and Singh, 2014).

**FIGURE 5:**
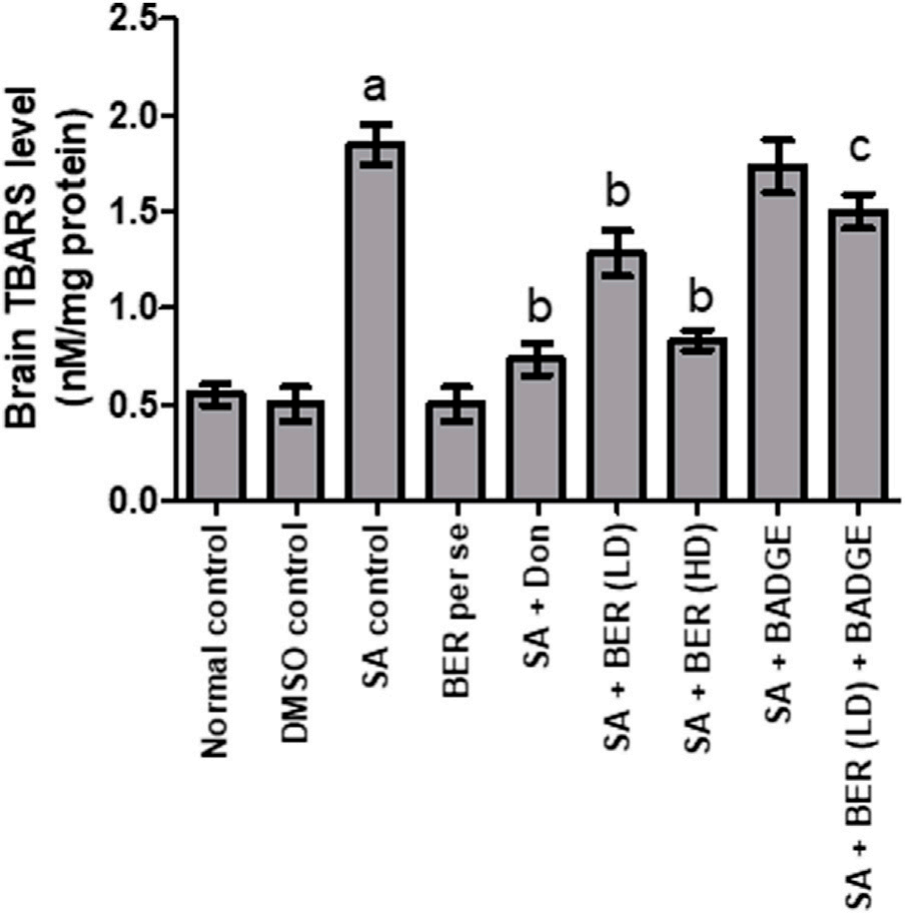
Effect of pharmacological interventions on brain TBARS level (nM/mg of protein). “DMSO, dimethyl sulfoxide; SA, sodium azide; Don, donepezil; BER, bergenin; BER (LD), bergenin low dose; BER (HD), bergenin high dose; BADGE, Bisphenol A diglycidyl ether” (Mehra et al., 2015). “Each group (*n* = 6) represents mean ± S.E.M, a = *p* ˂ 0.05 vs. normal control, b = *p* ˂ 0.05 vs. normal control, c = *p* ˂ 0.05 vs. SA control. One way ANOVA followed by Bonferroni’s multiple range test” (Sodhi and Singh, 2013b).

A notable increase in the brain GSH level and a marked reduction in the brain TBARS levels was indicated in animals upon sodium azide treatment (Figures 4, 5) while comparing with the control group revealing oxidative stress induction. Bergenin (30 mg/kg, p.o.) per se has not shown any noteworthy impact on TBARS and GSH levels when compared with the control (Figures 4, 5). On the other hand, we have observed a dose-dependent relief from oxidative stress via a reduction in the TBARS level and enhancement of GSH level (Kaur et al., 2018) when co-administered donepezil and bergenin in sodium azide-treated rats (Figures 4, 5). Administration of BADGE (30 mg/kg i.p.) along with bergenin (30 mg/kg p.o.) enhanced the oxidative stress levels in sodium azide-treated rats (Figures 4, 5) (Carey et al., 2022).

### 3.4 Effect on brain myeloperoxidase levels

**FIGURE 6:**
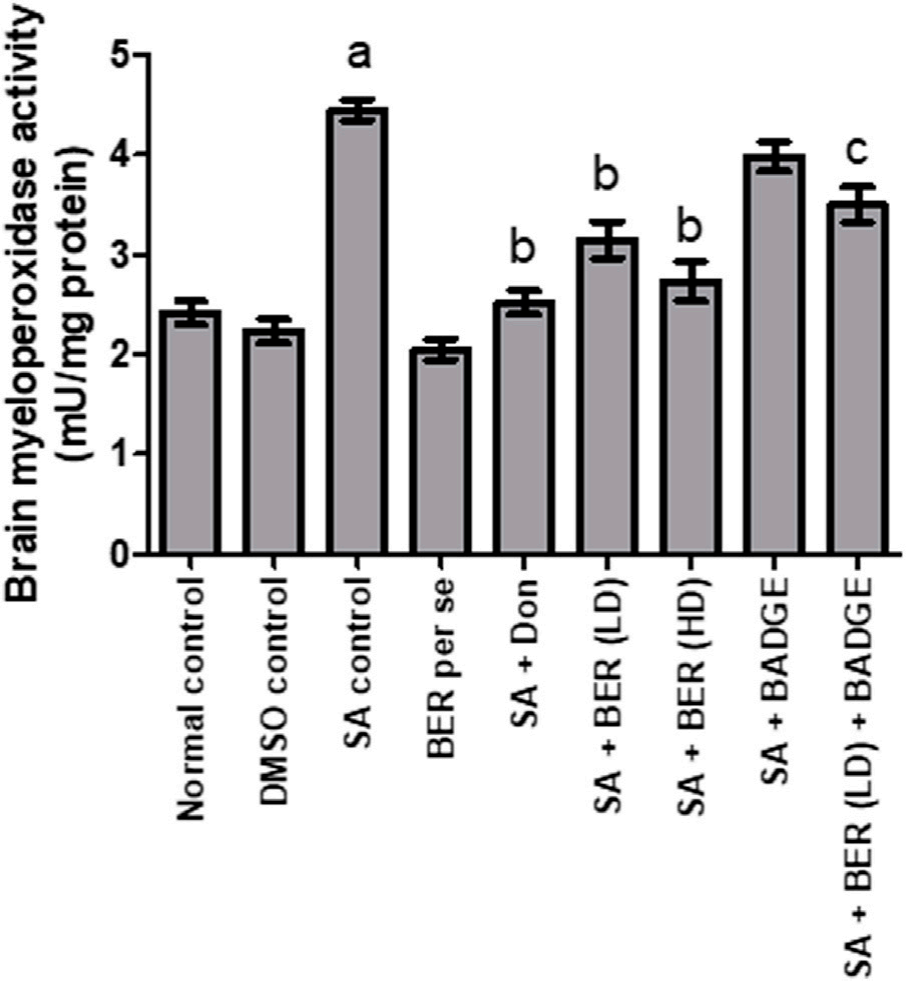
Effect of pharmacological interventions on brain MPO level (U/mg of protein). “DMSO, dimethyl sulfoxide; SA, sodium azide; Don, donepezil; BER, bergenin; BER (LD), bergenin low dose; BER (HD), bergenin high dose; BADGE, Bisphenol A diglycidyl ether” (Mehra et al., 2015). “Each group (*n* = 6) represents mean ± S.E.M, a = *p* ˂ 0.05 vs. normal control, b = *p* ˂ 0.05 vs. normal control, c = *p* ˂ 0.05 vs. SA control. One way ANOVA followed by Bonferroni’s multiple range test” (Sodhi and Singh, 2013b).

Treatment with sodium azide significantly enhanced the brain MPO activity in comparison to the control group (Figure 6) (Sodhi and Singh, 2013a). Bergenin per se produced no significant change in brain MPO levels (Sodhi and Singh, 2014) in contrast to the control group (Figure 6). Administration of donepezil and bergenin to sodium azide-treated rats significantly reduced the brain MPO activity indicative of anti-inflammatory effect (Figure 6). Co-administration of BADGE (30 mg/kg i.p.) with bergenin (30 mg/kg p.o.) to sodium azide treated rats markedly enhanced brain MPO activity (Figure 6).

### 3.5 Impact on pro-inflammatory cytokines

**FIGURE 7:**
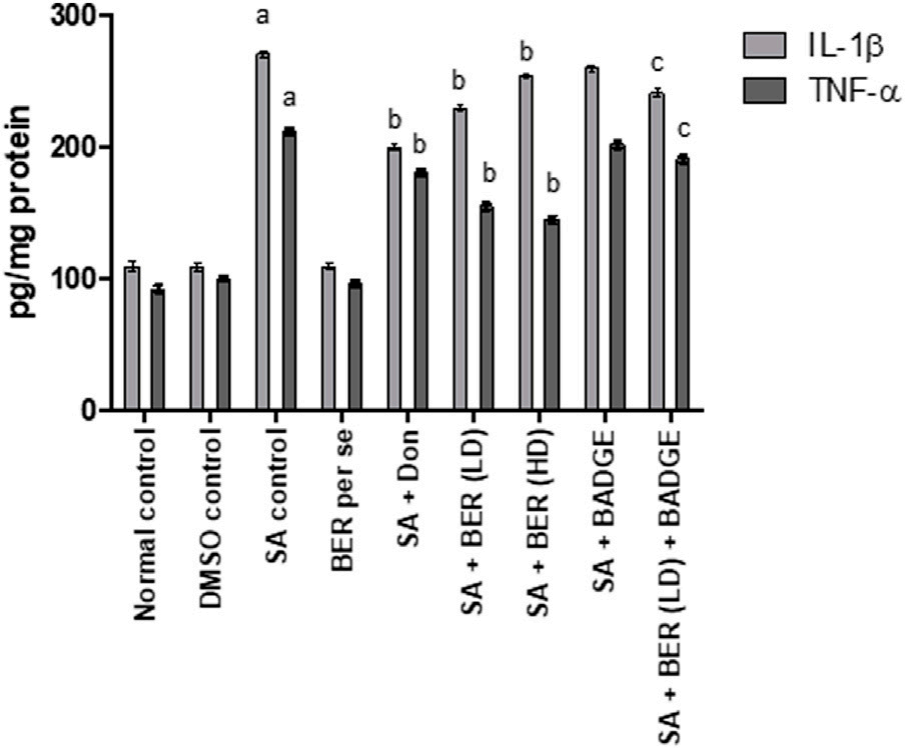
Impact of bergenin on brain cytokine levels (TNF-α and IL-1β) (pg/mg of protein). “DMSO, dimethyl sulfoxide; SA, sodium azide; Don, donepezil; BER, bergenin; BER (LD), bergenin low dose; BER (HD), bergenin high dose; BADGE, Bisphenol A diglycidyl ether” (Mehra et al., 2015). “Each group (*n* = 6) represents mean ± S.E.M, a = *p* ˂ 0.05 vs. normal control, b = *p* ˂ 0.05 vs. normal control, c = *p* ˂ 0.05 vs. SA control. One-way ANOVA followed by Bonferroni’s multiple range test”.

Treatment with sodium azide significantly enhanced the level of proinflammatory cytokines like IL-1 β and TNF-α when compared with the control group rats (Figure 7). Bergenin per se did not express any significant impact on of IL-1 β and TNF-α levels. Administration of donepezil and bergenin to sodium azide-treated rats significantly attenuated the IL-1 β and TNF-α levels. Administration of BADGE (30 mg/kg i.p.) along with bergenin (30 mg/kg p.o.) to sodium azide-treated rats markedly enhanced the expression of IL-1 β and TNF-α (Carey et al., 2022).

### 3.6 Impact on brain nitrite/nitrate level

**FIGURE 8:**
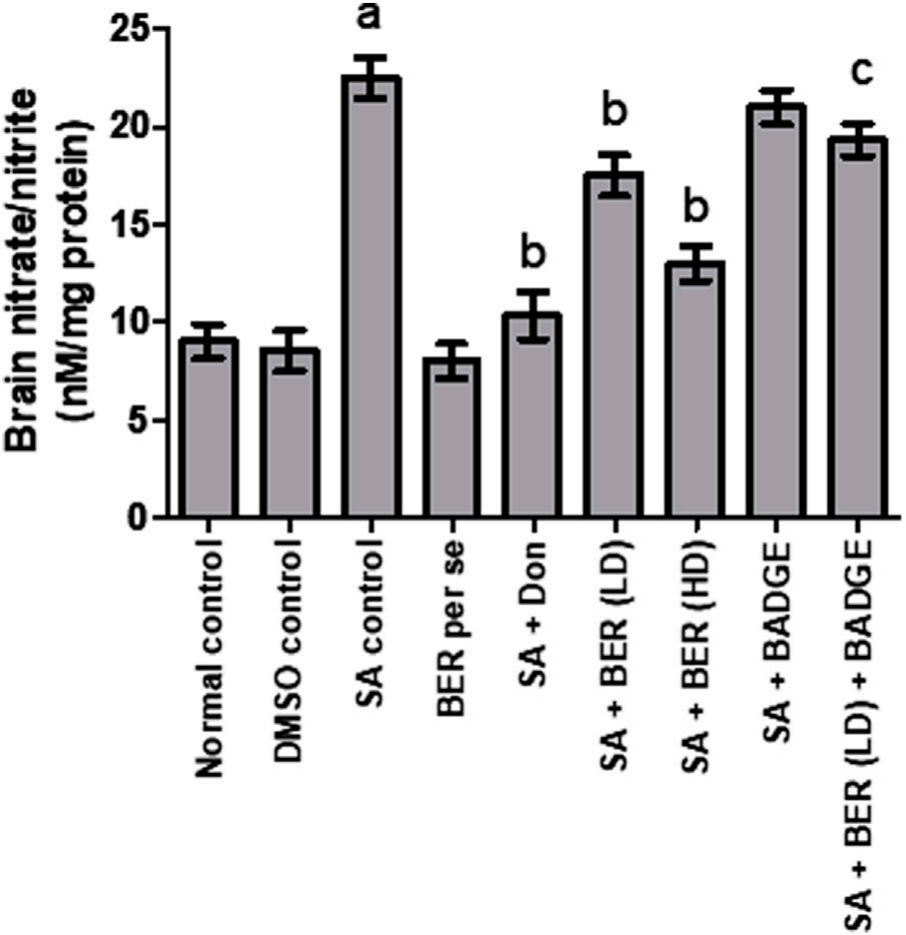
Effect of pharmacological interventions on brain nitrite/nitrate levels. “DMSO, dimethyl sulfoxide; SA, sodium azide; Don, donepezil; BER, bergenin; BER (LD), bergenin low dose; BER (HD), bergenin high dose; BADGE, Bisphenol A diglycidyl ether” (Mehra et al., 2015). “Each group (*n* = 6) represents mean ± S.E.M, a = *p* ˂ 0.05 vs. normal control, b = *p* ˂ 0.05 vs. normal control, c = *p* ˂ 0.05 vs. SA control. One-way ANOVA followed by Bonferroni’s multiple range test.”

Treatment with sodium azide significantly enhanced the brain nitrate/nitrite levels in comparison with the control rats (Figure 8). No significant alteration was detected in the brain nitrite/nitrate levels in the bergenin per se group while the comparison is made with the control rats’ group (Figure 8). Co-administration of donepezil (0.1 mg/kg/day, i.p.) and bergenin (30 and 60 mg/kg p.o.) to sodium azide rats markedly reduced the brain nitrate/nitrite levels when compared to the normal group (Figure 8). Concomitant usage of BADGE (30 mg/kg i.p.) with bergenin (30 mg/kg p.o.) to sodium azide-treated rats markedly enhanced brain nitrate/nitrite levels (Figure 8). These experiments were designed in a similar way to our previous experiments and other researcher’s results (Kaur and Sodhi, 2015; Ashwlayan, 2017).

### 3.7 Histopathological alterations in the brain

**FIGURE 9:**
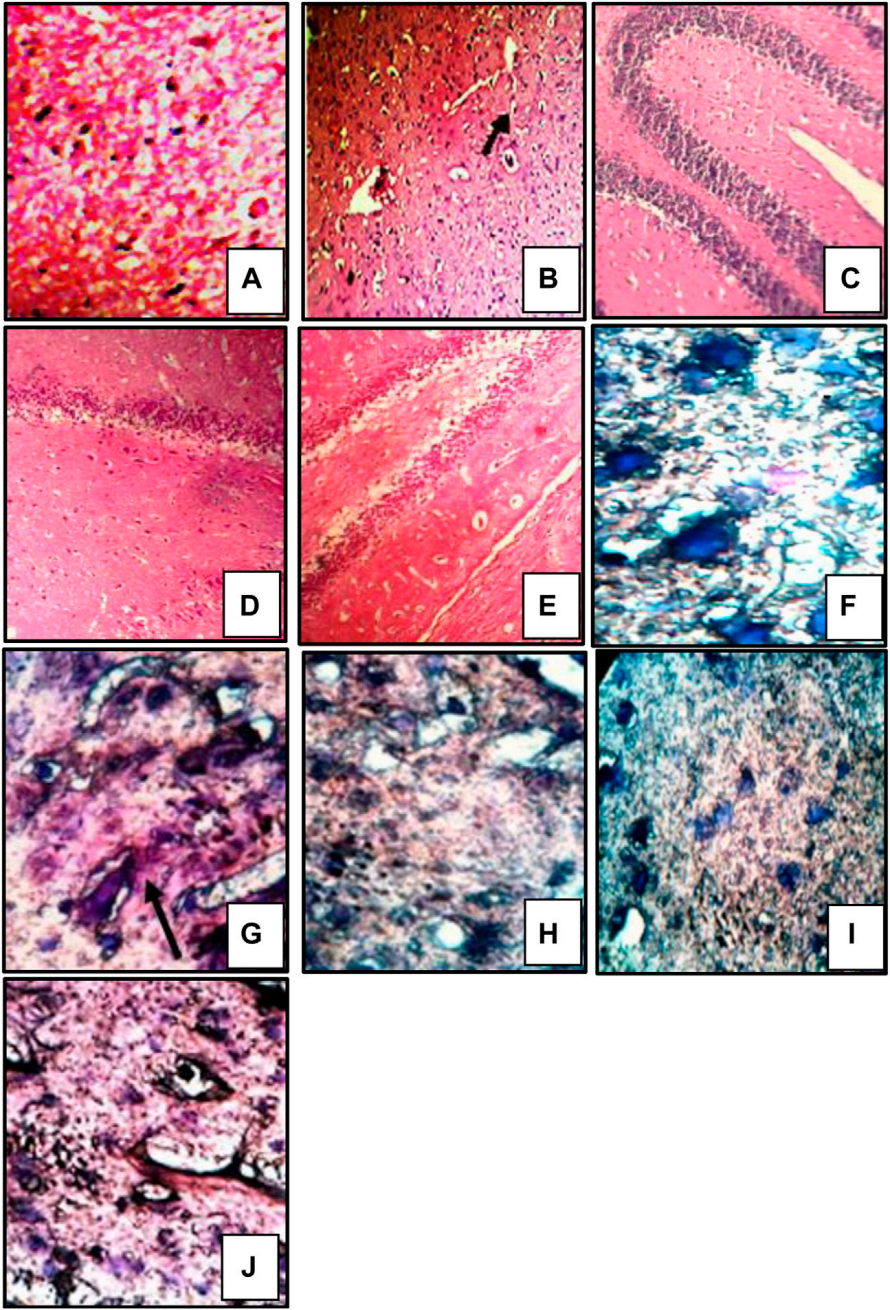
Histological sections of the brain were stained with HE staining, magnification 400 × **(A–E)**, and congo red staining, magnification 1,000 x; oil immersion **(I,J)** (Sodhi and Singh, 2014). “Control **(A)** shows normal histological features. SA-treated rat **(B)** showed focal as well as diffuse severe neutrophilic infiltration, congestion of blood vessels, and pericellular edema. Bergenin administered (30 and 60 mg/kg) SA-treated rats **(C,D)** respectively featuring mild neutrophilic infiltration and pericellular edema. BADGE administration (30 mg/kg) along with bergenin (30 mg/kg) to SA-treated rats **(E)** showed focal as well as diffuse severe neutrophilic infiltration. Control **(F)** congo red deposition was not detected. SA-treated rat **(G)** showing orange-red deposits of congo red. Bergenin administered (30 and 60 mg/kg) SA-treated rats **(H,I)** respectively showed significant congo red deposits. BADGE administration (30 mg/kg) along with bergenin (30 mg/kg) to SA-treated rats **(J)** showing orange-red deposits of congo red.”

Brain sections stained with hematoxylin and eosin exhibited severe neutrophil infiltration in sodium azide-treated rats indicative of neuroinflammation. Administration of donepezil and bergenin to sodium azide-treated rats produced a significant reduction in neutrophilic infiltration. Furthermore, sodium azide-treated rats demonstrated noticeable congo red staining observed as orange-red coloration signifying Aβ deposition (Sodhi and Singh, 2013b). Reduced Congo red deposition was observed in the donepezil and bergenin-administered sodium azide-treated rats (Figure 9) (Sodhi and Singh, 2013b).

## 4 Discussion

Alzheimer’s disease (AD) is a progressive multifarious neurodegenerative disorder characterized by senile plaques containing extracellular amyloidal protein deposits and intracellular neurofibrillary tangles (Kumar et al., 2015). Our study was undertaken to determine the defensive effect of bergenin on dementia induced by sodium azide in Wistar rats. We observed a significant development in cognitive parameters and biochemical alterations with the administration of bergenin indicative of its neuroprotective potential in certain markers of dementia (Neha et al., 2014). Further to gain evidence regarding the neuroprotective effect of bergenin we evaluated a series of parameters related to cognition, biochemistry, and histopathology. MWM test was conducted in the current study since it is amongst the commonly used well-established exteroceptive models to evaluate learning and memory in rodents including rats (Sharma and Singh, 2012; Sodhi and Singh, 2013b; Rani et al., 2015). Normal acquisition of memory in control untreated rats is denoted by a marked decline in escape latency time during acquisition trials and retrieval of memory is indicated by an enhancement of mean TSTQ in pursuit of the missing platform (Saraf et al., 2003; Kaur and Sodhi, 2015).

A sodium azide-induced experimental dementia model was used in our study to produce dementia of AD type. Chronic administration of sodium azide in rats produces pathological processes similar to that of AD, like reduction of mitochondrial complex IV activity (Nöldner, 2017). Further, the synthesis of the Aβ peptide through the distorted breakdown of the Aβ -protein precursor (AβPP) is pivotal in the pathology of AD. Disturbed processing of AβPP is observed during sodium azide treatment (Henriques et al., 2005). Sodium azide causes a diminution in mitochondrial cytochrome oxidase activity, an enhancement in the expression of amyloidogenic AβPP and Aβ 1–42 level, beta-site amyloid precursor protein cleaving enzyme 1 (BACE1), and attenuation in the neurotrophin’s expression in the region of hippocampus when administered to rats (Zhang et al., 2018). It has been demonstrated that there exists an association between “mitochondrial dysfunction and autophagy” in the course of AD’s pathology. Owing to inadequate digestion of oxidatively damaged macromolecules and organelles by autophagy, neurons progressively accumulate lipofuscin which could aggravate neuronal dysfunction (Moreira et al., 2010).

Results of our study indicate that administration of sodium azide (12.5 mg/kg for 5 days and 10 mg/kg for the next 9 days) resulted in the rigorous decline of spatial memory as signified by MWM test performance evaluated employing ELT and mean TSTQ (Morris, 1984). Moreover, a significant rise in acetylcholinesterase activity, nitrate/nitrite levels, and oxidative stress (shown by a significant increase in TBARS level and a decrease in GSH level) had also been observed (Kaur and Sodhi, 2015). Additionally, MPO activity was increased which is indicative of neuroinflammation. Donepezil acted as the positive control in the current investigation since donepezil is well accepted by the “U.S. Food and Drug Administration (USFDA)” to be efficacious for the management of moderate to severe levels of AD (Zhang and Gordon, 2018) and has been verified in our previous studies for experimental dementia (Sodhi and Singh, 2013a).

Bergenin is a colorless crystalline compound chemically occurring as a C-glucoside of 4-O-methyl gallic acid. It exhibits antiviral, antifungal, anti-inflammatory, antitumor, antitussive, antidiabetic, antiplasmodial, antihepatotoxic, antiulcerogenic, antiarrhythmic, and wound healing properties (Bajracharya, 2015). It has been reported that bergenin stimulates PPAR-γ, inhibits TGF-β, promotes autophagy, and reduces liver fibrosis by preventing hepatocyte necrosis and extracellular matrix development (Xia et al., 2020; Xiang et al., 2020). Several studies have proposed the anti-inflammatory and anti-oxidative potential of bergenin in different pathological states (Oliveira et al., 2019; Villarreal et al., 2020). Bergenin has been identified as a PPAR-γ agonist to promote nuclear translocation and transcriptional activity of PPAR-γ. It also augments the expression of mRNA related to CD36, LPL, and ap2 (Dave et al., 2012). In addition, it has also been depicted that bergenin enhances the expression of Sirtuin 1, inhibits the NF-κB-p65 acetylation, and increases NF-κB-p65 and IκBα association (Wang et al., 2018). Another study indicated that bergenin and its analogs have an inhibitory effect on BACE1 and hence may be useful in Alzheimer’s disease (Kashima and Miyazawa, 2013). It has been documented that the PPAR response element is located in the BACE-1’s promoter region, and the binding of PPAR to the response element suppresses the expression of BACE-1 and hence restrains Aβ synthesis (Heneka et al., 2005).

In this present study, we observed that the administration of PPAR-γ agonist, bergenin (30 mg/kg and 60 mg/kg; p.o.) for 2 weeks to sodium azide-treated rats prominently improved the cognitive deficiencies evoked by sodium azide. Bergenin treatment led to a noteworthy decrease in fourth-day ELT and an increase in the fifth-day TSTQ in the MWM test in contrast to the sodium azide-treated group (Kumar and Singh, 2017). Administration of bergenin caused a noteworthy decrease in brain AChE activity, brain TBARS, brain nitrite/nitrate level, and brain MPO activity which were increased due to sodium azide administration, and an increase in GSH level was also observed (Gulati and Singh, 2014). Furthermore, bergenin administration reduced the histopathological alterations, exemplified by low neutrophilic accumulation, and pericellular edema, which recommends a noticeable protective and anti-inflammatory effect against the toxic effects of sodium azide (Sodhi and Singh, 2014).

PPAR—γ did contribute an important and vital role in modulating gene expression associated with multiple diseases which include obesity, diabetes, and cancer (Janani and Ranjitha Kumari, 2015). Study indicates that PPAR- γ stimulation mitigates the inflammation associated with chronic and acute neurological insults with the major mechanism involving PPAR- γ (Kapadia, 2008). PPAR-γ-induced neuroprotection may be an outcome of avoidance of microglial activation and inflammatory cytokine and chemokine expression. PPAR-γ impedes NF-κB action by hindering NF-κB nuclear translocation and challenging NF-κB for co-activators, therefore, holding potential for the treatment of neuroinflammatory disorders (Chen et al., 2012). Wang and the team conducted another study on APP/PS1 mice treated with a natural PPAR-γ agonist, astragaloside IV, which increased the activity of PPAR-γ and inhibited BACE1 thereby decreasing Aβ levels and neuritic plaque formation significantly (Wang et al., 2016).

In the present investigation, BADGE, a PPAR-γ antagonist was administered to the rats to verify whether it reverses the neuroprotective effects produced by bergenin. Administration of BADGE to bergenin-treated sodium azide rats significantly reversed the protective biochemical and histopathological alterations produced by bergenin. A marked deterioration of memory was also indicated. Hence it may be worth suggesting that bergenin may elicit neuroprotective effects in AD-type dementia owing to its memory restorative, antioxidative, anti-inflammatory, and anticholinesterase activity. It may also be suggested that neuroprotective effects produced by bergenin may involve the activation of PPAR-γ receptors.

## 5 Conclusion

Our study provides evidence that bergenin improves cognition and memory in sodium azide-induced experimental dementia by virtue of its neuroprotective, memory restorative, anti-cholinesterase, anti-oxidative and anti-inflammatory activity accompanied by a decrease in brain neutrophil infiltration (Sodhi and Singh, 2013a). It may also be concluded that bergenin produced its beneficial action through the activation of PPAR-γ receptors. Nevertheless, further studies are needed to confirm the neuroprotective activity of bergenin through PPAR-γ activation.

## Data Availability

The original contributions presented in the study are included in the article/Supplementary Material, further inquiries can be directed to the corresponding authors.
